# Modeling and forecasting Saudi banking stability using ARIMA and exponential smoothing technique

**DOI:** 10.3389/frai.2026.1702414

**Published:** 2026-02-13

**Authors:** Abdulaziz Alnajjar, Hamzeh F. Assous, Hazem Al-Najjar

**Affiliations:** 1 Department of Finance, School of Business, King Faisal University, Al-Ahsa, Saudi Arabia; 2 Department of Computer Engineering, College of Engineering, Al-Balqa Applied University, As-Salt, Jordan

**Keywords:** ARIMA models, asset quality, banks’ wealth, capitalization, economic growth, forecasting models, liquidity, profitability

## Abstract

This research examines the key factors influencing the financial stability of Saudi banks by developing an optimal stepwise linear regression model. The research uses financial information gathered from 11 Saudi banks over the period 2014–2021. Six categories for key performance indicators (KPIs) which consist of profitability, liquidity, asset quality, capitalization, bank size and economic growth are included in the model. The *Z*-score is used as its dependent variable for all stability measures. A model with the lowest standard error should be selected as the best explanatory model among all options while also maintaining the highest adjusted R-squared value. The findings showed that the chosen model has the lowest standard error around (7.209) and the highest adjusted R-squared (71.3%), The study demonstrates that NII1 ratio and CAR statistics alongside bank asset size (log of assets) produce positive effects on stability yet the stability declines when banks use investment ratio statistics or loan impairment ratio indicators. Economic growth (GDP) shows no significant influence. The second phase of this research uses ARIMA and exponential smoothing models which are selected to produce *Z*-score predictions through 2030. The chosen forecast validation metrics include RMSE, MAE, MAPE and E-square. The standardized forecasts enable banks to compare resulting data with each other. The financial performance data shows different trends. Studies indicate that Arab National Bank and National Commercial Bank will provide consistent financial outcomes. Saudi Investment Bank and Bank Al - Jazira have moderate trends with high forecast precision. Al Rajhi Bank, Samba Financial Group and Saudi British Bank continue to operate steadily. The empirical findings offer support to stakeholders and regulatory authorities in decision-making processes that enable alignment with the Vision 2030 objectives.

## 1 Introduction

Banks are a crucial component in promoting economic growth as it is move them act as intermediaries between those who have excess financial resources and those who are facing financial deficits. Due to their considerable influence on the economy, they are regarded as the most efficient financial organizations. Since it affects their capacity to make money and strengthen the economy as a whole, the stability of banks is a key determinant of their success. Stable banks are better able to absorb shocks and maintain continuous development, which benefits the banking industry along with the entire economy. A substantial association between a bank’s efficiency score and other factors, such as liquidity, managerial effectiveness, cost efficiency, and capital sufficiency has been demonstrated by academic research (Hewaidy et al., 2020; Al-Najjar and Assous, 2021; Assous, 2022). In the years after the share market crash of 2008, the subject of banking stability received a lot of attention.

Despite this, there have not been many studies looking at how crucial bank stability is for the whole economy. The idea of financial stability has been a top concern for central banks and other financial authorities in recent years (Mabkhot and Al-Wesabi, 2022).

The Kingdom of Saudi Arabia is the ninth-largest economy worldwide and a major producer of oil. The kingdom is also a G20 member and is strategically situated at the crossroads of vital economic lines that pass through three continents. Saudi Arabia offers numerous growth opportunities for corporate progress in a variety of economic industries due to its wealth of natural resources and forward-thinking economy. The Saudi Vision 2030 involves a number of economic changes that, leveraging the kingdom’s strategic assets, have already generated new commercial prospects. With the ultimate objective of making Saudi Arabia an appealing and stimulating investment destination for investors across all industries, these measures are promoting economic development and diversification (Al-Najjar and Assous, 2021; Al-Najjar, 2022). The Saudi Arabia financial system has been steady over the past 4 decades. However, the banking industry has faced a number of challenges, including the latest COVID-19 epidemic, recent economic downturns, instability and volatility in the global financial markets, and worldwide financial crises (Assous et al., 2020; Assous and Al-Najjar, 2021; Assous, 2022). Notwithstanding the above-mentioned challenges, banking institutions in Saudi have maintained their relative dominance and built a robust capital base, thus avoiding significant financial turbulence.

Currently, banks operating in Saudi Arabia are in a favourable position in terms of capitalization, quality of assets and technological infrastructure, and therefore in a position to play a pivotal role in the international and regional financial markets. As one of the largest banking sectors in the GCC, Saudi banks had 11 public listings as of the end of 2021 and based on the latest merger between National Commercial Bank and Samba Group, the number decreased to 10 listings as of the end of 2022. However, the banking sector in Saudi Arabia has seen a significant influx of foreign bank branches in recent years and the number of incoming foreign banks is expected to increase. This is due to many economic and financial reforms lately adopted by Saudi Leaders (Assous and Al-Najjar, 2021).

On the other hand, Saudi banks are confronting numerous obstacles. These challenges began with the surge in oil prices following the 2008 subprime crisis and were compounded by political instability in the MENA region that began in late 2010 with events in Tunisia and spread rapidly to Yemen, Syria, Egypt, and Libya. Moreover, COVID-19 pandemic at the end of 2019 has had significant consequences, including high levels of inflation and sharp fluctuations in interest rates. The above mentioned challenges have had a noticeable impact on the economic performance of the Saudi Arabia and on the global growth trajectories (Mabkhot and Al-Wesabi, 2022).

Central banks have each year published financial stability reports that are designed to maintain the soundness of banking systems and financial institutions. In recent years, a slowdown in growth has affected Saudi Arabia and other countries in the Gulf Cooperation Council (GCC), with equity markets reaching a low in early 2016, during the period of low oil prices at roughly USD 30 per barrel. Given the pre-eminent role of oil receipts in the national economy, there is a strong nexus between crude prices and macro-financial stability. As a result, drops in oil prices can trigger a range of financial system problems, including liquidity problems and asset quality decline (International Monetary Fund, 2015). These developments point to the importance of banking stability and the debt capital market, especially as the economy becomes more dependent on them. Nevertheless, the debt capital market in Saudi Arabia is relatively under-developed. Improving the domestic capital market would result in more resourceful capital allocation and better risk distribution, which would boost access to financing for private-sector infrastructure projects (International Monetary Fund, 2018).

Despite the increased literature on banking stability, the extant studies on the topic in Saudi Arabia and the Gulf Cooperation Council as a whole tend to focus on cross-country comparisons or short-term measures, hence often overlooking bank-level heterogeneity and the processes of long-term stability (Mabkhot and Al-Wesabi, 2022; Ghassan and Fachin, 2016). There is little empirical evidence that merges both Islamic and conventional banks into a unified analytical model (Nosheen and Rashid, 2020; Bourkhis and Nabi, 2013), and very few studies simultaneously utilize econometric analysis and prospective forecasting of the evolving economic conditions, such as the Vision 2030 reforms (Al-Najjar, 2022; Assous, 2022).

This work seeks to fill the identified gaps by conducting a systematic research on the determinants of banking stability in the Kingdom of Saudi Arabia by employing the use of the *Z*-score model (Borio, 2006; Goodhart, 2006). Such variables as capital adequacy, profitability, bank size, asset quality, liquidity, investment intensity, and economic growth are ranked highly in the analysis (Kamran et al., 2019; Sufian and Chong, 2008; Regehr and Sengupta, 2016; Ghenimi et al., 2017; Suljić Nikola et al., 2022). The study utilizes a set of time-series extrapolation methods to project stability scenarios to 2030 (Assous et al., 2020; Assous and Al-Najjar, 2021), and thus, provides an overall, future-oriented opinion that helps understand the healthiness of the financial sector and how to formulate corresponding policy objectives.

## 2 Literature review and hypotheses development

This section gives an overview on stability stated through the *Z*--score, identifies the main factors determining stability and develops a theoretical framework and the respective hypotheses.

### 2.1 Stability overview

This section gives an overview of the *Z*-score methodology and compares conventional banking and Islamic banking in evidence of financial stability.

#### 2.1.1 *Z*-score model

The International Monetary Fund (IMF) lists a set of key indicators for assessing the robustness, reliability and stability of a banking institution. These indicators include profitability, which is measured using the return on assets (ROA); quality of assets, measured using the ratio of non-performing loans (NPLs) to total loans; and capitalization, which is measured using the capital adequacy ratio (Pointer and Khoi, 2019; Nugroho et al., 2020). Theoretical and empirical studies (Borio, 2006; Goodhart, 2006; Di Giorgio and Rotondi, 2011) suggest that financial instability has consequences beyond the boundaries of the financial system, such as in the equity markets, debt markets, banking institutions and payment and settlement mechanisms and can create large disturbances in the real economy. Such disturbances occur as there are abrupt fluctuations in a variety of financial prices and costs.

Unexpected shocks arising within the financial system can disrupt the economy’s steady development, harm the confidence of individuals and firms and inhibit the efficient allocation of wealth. Furthermore, the financial system may be inadequate in terms of allocation or reallocation of risks among its stakeholders (Ghassan and Fachin, 2016). Financial system stability is characterized by a state where instruments responsible for price determination, capital allocation, and risk management function well and, in turn, foster economic growth. A well-built stable financial system in turn represents benefits, protection from negative shocks, and at the same time, ensures the overall stability and resilience of the system (Rashid and Jabeen, 2016; Ajizah and Widarjono, 2023).

Financial stability is concerned with issues of instability and volatility that primarily affect financial institutions, rather than non-financial institutions (Issing, 2003). Building on this, Mabkhot and Al-Wesabi (2022) define financial stability as the capacity of financial organizations to withstand economic tremors, absorb the consequences of financial crises, and evaluate and control risks. As stated in Allen and Wood (2006), one way to explain banking stability is by contrasting it with the characteristics of instability. In this regard, banking stability can be viewed as periods without significant instability. Likewise, financial stability brings up the ability of the financial system, which includes banks, other financial intermediaries, financial markets, and the infrastructure supporting them, to endure shocks and financial crises (Nosheen and Rashid, 2020).

The banking *Z*-score model is a commonly used method for evaluating the general health and stability of banks along with their creditworthiness, risk of bankruptcy, and financial stability. Numerous studies have shown that it does work. As explained in Bourkhis and Nabi (2013), the *Z*- score compares the market value of the assets of a bank to its book value of liabilities, and is a distance to default measure. In order to measure the financial health of the Islamic banking sector effectively, they use the *Z*-Score methodology, the higher the *Z*-Score the less is the probability that the bank will go bankrupt and the more is the overall stability of the bank. Numerous prominent researches have adopted *Z*-score, which is obtained by taking the sum of return on assets (ROA) and equity-to-assets and dividing the same by standard deviation of return on assets (ROA) to measure the financial stability of banks. This formula is a key tool is the assessment of the soundness of banks (Fiordelisi and Mare, 2014; González et al., 2017). The *Z*-score measures the number of standard deviation changes in a bank’s ROA which a bank is able to withstand until its protection of capital stops. In effect, it measures the number of times that the equity cushion of a bank can absorb losses before defaulting on its debts.

As a result, a greater *Z*-score denotes a decreased probability of bank default (Miah and Uddin, 2017).

#### 2.1.2 Islamic versus conventional banking and financial stability

The co-existence of Islamic and conventional banks, especially in the dual banking systems like Saudi Arabia, is increasingly featured in the literature on the topic of financial stability. Conventional banks rely on the interest-based intermediation where the Islamic banks are governed by the Shariah law that forbids riba, and prioritizes profit-and-loss sharing, asset-backed financing, and risk-sharing agreements. These differences in structures affect risk profiles, capital structures, liquidity management, and stability of the income of banks (Čihák and Hesse, 2010; Beck et al., 2013; Bourkhis and Nabi, 2013).

Despite institutional differences, empirical studies show that Islamic and conventional banks are largely impacted by similar factors of financial stability. Examples of this include capital adequacy, which increases the loss absorption capacity of a bank; profitability, which generates internal capital; and a larger bank size, which provides benefits of diversification, all of which increase financial stability in both bank types (Rajhi and Hassairi, 2013; Miah and Uddin, 2017; Adusei, 2015). On the contrary, a decline in asset quality coupled with an increase in liquidity risk are correlated to a high likelihood of insolvency, regardless of whether the banking system is operating under a contractual regime (Ghenimi et al., 2017; Zaghdoudi, 2019; Kharabsheh and Gharaibeh, 2022).

Consequently, the *Z*-score proves to be a widely accepted index of banking stability that can be applied to Islamic and conventional banking institutions. It assesses the risk of insolvency using a combination of profitability, capital adequacy, and earnings volatility measures and omitting interest-based measures (Čihák and Hesse, 2010; Karim et al., 2018; Kumar, 2022). Such an integrative approach is of particular relevance in the context of Saudi Arabia where Islamic and conventional banks coexist under a single regulatory and supervisory framework and are facing the same macroeconomic factors (Ghassan and Taher, 2013; Mabkhot and Al-Wesabi, 2022).

### 2.2 Key determinants of stability

#### 2.2.1 Banks’ wealth

Leveraging economies of scale allows larger banking institutions to spread their fixed costs across a larger number of assets and, in doing so, reducing their average costs and increasing their profitability (Regehr and Sengupta, 2016). The *Z*-score of a bank is significantly affected by its total assets & capital adequacy ratio (CAR), which shows that high values of these variables may increase the stability of banks. Accordingly, banks are required to intensify their assets management to meet the role of the financial intermediary that supports the growth of the public economy (Widarjono, 2020). These conclusions are consistent with the research results of Ajizah and Widarjono (2023) and Rajhi and Hassairi (2013). Slimen et al., (2021) show that bank size has a significant positive impact only on short term stability and Beck (2008) finds a positive correlation between bank’s size and the stability of bank as measured by L-Z score. A study of Malaysian banks between 2005 and 2010 by Rahim et al. (2012) confirms the positive relationship between the financial stability of banks and bank size, as measured by total assets. Finally, there is research by Čihák and Hesse (2010) that finds financial stability is positively affected by both governance practices and bank size.

#### 2.2.2 Asset quality

As Ledhem and Mekidiche (2020) argued, the asset quality is a key factor in determining the banking stability of Islamic banks. High asset quality is linked to both strong profitability and financial stability, and is often assessed by examining the ratio of non-performing loans to total loans. Kumar (2022) has demonstrated that the quality of a bank’s loan portfolio can be effectively measured using the NPL ratio. A higher NPL ratio increases a bank’s vulnerability and puts the banking system at greater risk, as it can lead to a depletion of the bank’s capital. Suljić Nikola et al., (2022) suggested that non- performing loans should be handled to reduce their negative impact on the banking industry since they have a negative influence on a bank’s performance and are an unwanted cost. Chai et al. (2022) investigated the impact of different risk types on the stability of 15 Pakistani banks for the period from 2009 to 2020. The results show that there is a negative relation between credit risk and liquidity risk and bank stability and the funding risk does not have any statistically significant impact on stability.

Maritsa and Widarjono (2021) confirm that the long-term impact of continuous non-performing loans on Islamic banking sector can result in significant losses. Adusei (2015) used quarterly data from 2009 to 2013 and found a very significant negative relationship between credit risk and stability of banking sector in Ghana. Using a sample of forty-nine nations from 2006 to 2013, Ghenimi et al. (2017) conclude that credit risk is an important source of bank instability. Zaghdoudi (2019) provided evidence, based on the Tunisian conventional banking sector, that credit risk has a negative impact on stability. Kharabsheh and Gharaibeh (2022) discovered that credit risk has a significant negative effect on the stability of commercial banks in Jordan. Prima Sakti and Mohamad (2018), argued that Islamic banks demonstrate higher asset quality, which can be attributed to several factors, including lower loan loss provisions and a reduced number of non-performing loans. Ajizah and Widarjono (2023) demonstrated that nonperforming loans negatively affect the performance of Islamic banks in Indonesia.

#### 2.2.3 Liquidity

The assessment of a bank’s liquidity can be determined by calculating the ratio of current assets to gross assets (Younes, 2022). While, Ledhem (2022) demonstrated that Islamic banks’ financial stability is weakened by liquidity risk. This is due to the fact that high intermediation margin could potentially have adverse consequences on central banks’ stability. As indicated by Sun et al. (2017) if liquidity risk forces Islamic banks to choose higher intermediation margins as a kind of compensation, this adverse outcome may occur in their case. This may raise borrowing costs for other Islamic banks or for sukuk markets as a result. They continued by saying that the existence of liquidity risk could be detrimental to stability as it might encourage banks to choose higher intermediation margins as a form of compensation, predominantly when they are running low on cash and risk incurring debt cost from other banks or financial markets. Liquidity risk is therefore expected to negatively affect the financial stability of Islamic institutions.

#### 2.2.4 Profitability

Ledhem and Mekidiche (2020) found that a correlation exists between asset quality and profitability, where sound asset quality is linked to elevated profitability and robust financial stability. Jiménez et al. (2013) observed a link between profitability, stability, and competitiveness and noted that as competition among US banks intensified, so did risk-taking tendencies.

In practice, the banks take on a greater risk in the face of fierce competition, as such competition narrows the profitability and may undermine financial stability. Concurrently, Ledhem (2022) also reported that asset growth has a negative impact on profitability, which could further reduce the stability of the Islamic banks.

Peura and Keppo (2006) showed that profitability and economic growth are basic determinants for determining a sustainable banking system, which in turn has positive impact on the stability of commercial banks operating in the Middle East and North Africa (MENA) region. Sun et al. (2017) found an inverse relationship between managerial quality and institutional stability that suggests that high quality management practices may have the counterintuitive effect of reducing profit levels, and consequently, financial stability. Elsayed et al. (2023) argued that the sources of finance used by Islamic banks are that they supplement both profit and resiliency, and allow these institutions to insulate the economy by absorbing losses during periods of economic turbulence.

#### 2.2.5 Capitalization

Beck et al. (2013) understood that the concept of banking stability has been formalised by various quantitative indicators, in particular the *z*-score. They found substantial differences between conventional and Islamic banking institutions, in particular, they noted that strong capitalisation in Islamic banks was related to comparatively good performance and resilience during the global mortgage crisis. Ghassan and Taher (2013) reported that higher *z*-score is associated with reduced likelihood of insolvency of the bank, with the index increasing with increase in capitalisation.

According to Ledhem and Mekidiche (2020), Capital Adequacy Ratio is calculated by dividing the total amount of Tier 1 and Tier 2 capital by risk-weighted assets. A higher ratio indicates that Islamic banks have adequate capital relative to their risk exposure and by this means they increase stability. Both Ledhem and Mekidiche (2020) and Karim et al. (2018) reported that the Capital Adequacy Ratio have a positive impact on financial stability of Islamic banks. Kassem (2022) proved, through the adopted models, that there is a statistically significant and positive relationship between the capital requirements and the bank stability. Moreover, the study recorded significant positive links between the dummy variables of Basel II and Basel III, indicating that stricter capital requirements act as a buffer against unexpected losses so that stability is reinforced.

Albaity et al. (2021) and Beck et al. (2013) found that the implementation of capital regulations leads to an increase in bank stability. In a related investigation, Kharabsheh and Gharaibeh (2022) investigated factors influencing the financial stability of Jordanian commercial banks using annual data from 2011 to 2018 and an aggregated effects model and concluded that SME financing and adequate capital have a positive and significant effect on the stability. Analysing Pakistani data spanning 2007 to 2016, Kamran et al. (2019) reported that increase in Capital Adequacy Ratio improves the financial stability of commercial banks although excessively high CARs have a detrimental effect on stability. Daoud and Kammoun (2020) in a study of data from twenty-two countries also found a positive impact of capital adequacy on bank stability.

#### 2.2.6 Economics’ growth

The concept of economic growth can be made to understand through Robinson’s (1952) demand following hypothesis. The relationship between banking and economic growth suggests that, as the economy grows, there is more demand for banking services, and thus more banking activity in the economy. As a result, this escalation can lead to increased loan volumes and increased customer deposits, which can strengthen the bank’s profitability and stability (Sufian and Chong, 2008). Therefore, Ledhem and Mekidiche (2020) confirmed a positive relationship between economic growth and the financial stability of Islamic banks. As a result, it can be inferred that as economic growth increases, so does the financial stability of Islamic banks. Kassem (2022) argued that economic growth, measured by ΔGDP, and the stability of commercial banks shows a positive and significant correlation. Many studies on financial stability have provided evidence that there is a positive correlation between bank stability and real GDP (Beck et al., 2013; Čihák and Hesse, 2010; Ghassan and Fachin, 2016; Miah and Uddin, 2017; Mabkhot and Al-Wesabi, 2022).

For the period 2006–2009 in Pakistan, a study by Shahid and Abbas (2012) was conducted and they found that financial stability was positively correlated with GDP growth. Rahim et al. (2012) examined the factors that contributed to the financial stability of institutions in Malaysia from 2005 to 2010. Their empirical findings revealed that both bank size and GDP growth enhance financial stability. While Elbadri and Bektaş (2017) conducted a study on financial stability in Turkey over the period from 2006 to 2013 considering both internal and external determinants. Their findings revealed that financial stability is positively affected by the cost to income ratio, inflation, and GDP growth.

### 2.3 Theoretical framework and conceptual model

This study uses a financial stability model whereby financial stability, measured by the *Z*-score, depends on bank-specific factors, and macroeconomic variables. Hypotheses postulated to have positive impact on stability are capital adequacy (H1), profitability (H2) and bank size (H3), which can be explained by the increased loss-absorbing capacity, income-generating capacity, and diversification benefits. On the other hand, the risk of asset quality (H4) and the intensity of investment suggesting reduced liquidity (H5) are predicted to have a negative impact on stability because a high level of credit risk and more limited liquid buffers increase the insolvency risk. The economic growth (H6) is theoretically expected to strengthen banking stability through increasing the repayment ability of the borrowers and also enhancing the overall macroeconomic conditions. This framework assumes a one-way causal relationship between these determinants and the stability of banks.

### 2.4 Hypothesis development

The research uses established scholarly findings to create hypotheses about bank stability in Saudi banks using the *Z*-score model. Empirical research examines the stability of Saudi banks by focusing on six widely recognized determinants that include banks’ wealth, asset quality, liquidity, profitability, capitalization and economic growth. The hypotheses rely on academic research results which identify patterns of stability influence between positive and negative factors in banking sectors.

*H1*: There is a significant and positive effect of banks’ wealth on Saudi banking stability.

The total assets or size measurement of banks acts as a critical determinant for financial stability enhancement. Firstly banks that operate at a larger scale benefit from reduced costs and wider investment possibilities and multiple funding channels. Regehr and Sengupta (2016) demonstrate the same findings as Widarjono (2020) and Ajizah and Widarjono (2023) when showing that financial stability can be predicted by total assets and bank size due to their robust revenue streams and extensive financial buffers. Within the Saudi Arabian banking sector wealthier banks demonstrate better stability and resilience in operations.

*H2*: There is a significant and negative effect of asset quality on Saudi banking stability.

The financial soundness of an entity depends strongly on its asset quality which is assessed through non-performing loans (NPL) ratio measurements. The existence of high NPL ratios indicates below-average asset value and increased default probabilities that create bank failures and profitability declines. Research from Ledhem and Mekidiche (2020) and Kumar (2022) along with Ghenimi et al. (2017) demonstrates that deteriorating asset quality as a sign of rising credit risk diminishes banking stability. Regionally and globally established research predicts the correlation between poor asset quality in Saudi banks and their lower *Z*-scores as indicators of increased instability.

*H3*: There is a significant and negative effect of lower liquidity (high investment intensity) on Saudi banking stability.

Bank performance gets strongly affected by the risk associated with liquidity. Insufficient liquid cash reserves in banks make them prone to market disruptions while increasing their funding expenses. Sun et al. (2017), Ledhem (2022), and Younes (2022) found that banking instability increases when liquidity risk reaches high levels which affects Islamic banking systems without sufficient lender-of-last-resort support. The hypothesis suggests that inadequate liquidity management in Saudi Arabia weakens financial stability since high liquidity acts as a negative influence in *Z*-score analysis.

*H4*: There is a significant and positive effect of profitability on Saudi banking stability.

The level of profitability protects financial institutions from loss and provides evidence about management effectiveness. Territorial banks may improve their reserves accumulation without compromising investor confidence in their stability. A significant positive relationship between the stability of the banking sector and the profitability of the banking sector can be observed from the research of Peura and Keppo (2006), Elsayed et al. (2023), and Jiménez et al. (2013). The banking institutions of Saudi Arabia are usually able to survive unfavorable financial conditions because their profitability is dependent on oil-dependent economic cycles.

*H5*: There is a significant and positive effect of capitalization on Saudi banking stability.

Capitalization shows the loss resilience of a bank when evaluated through the Capital Adequacy Ratio (CAR). Banks with sufficient capital have a lesser likelihood of facing financial collapse. Various banking systems show that there is a positive correlation between institutional stability and strong capital bases as is documented by Beck et al. (2013), Kassem (2022), and Kamran et al. (2019). Accordingly, the hypothesis assumes that high capital levels within the banks in Saudi increases financial stability by increasing the resistance to shocks.

*H6*: Economic growth (GDP) does not exert a statistically significant effect on Saudi banking stability.

Economic growth is a macroeconomic determinant of the health of the financial sector. A growing economy leads to increased demand for banking services, better performance of the loans and an increase in the deposit levels. Empirical research by Kassem (2022), Sufian and Chong (2008), and Beck et al. (2013) proves the claim that the stability of banks is positively correlated with the growth of GDP. In the case of Saudi Arabia, which has a significant programme of economic diversification under the role of Vision 2030, the growth of economic activity is expected to help positively with the stability of the banking system.

These six hypotheses provide the analytical base for the analysis of the determinants of banking stability in Saudi Arabia that can subsequently help to assess the empirical robustness of the *Z*-Score as a composite risk indicator with respect to various financial dimensions.

## 3 Research methodology

### 3.1 Data and description

The first objective of this work is to examine the key determinants that underscore the stability of banking institutions in Saudi Arabia and consequently see which linear regression is the most appropriate. The paper ends with the selection of ARIMA and exponential smoothing methods to create *Z*-score forecasts out through 2030. Data for this investigation have been extracted from annual financial statements of eleven Saudi banks for the period of 2014–2021. Because the merger of Samba Bank with National Commercial Bank excluded Samba’s financial figures from the financial year of 2021, those observations did not exist. Missing entries were treated with linear interpolation and seasonal adjustment procedures which is a standard technique in financial time-series analysis. This method preserves the temporal structure of the data while minimizing estimation bias and preventing artificial volatility in the *Z*-score series.

The explained variable, which measures the stability of the banks, was identified via the banking *Z*-score, a widely used index that pertains to the banking sector worldwide.

This study categorizes the primary determinants into different groups, including banks’ wealth, asset quality, liquidity, profitability, capitalization, and economic growth. The research methodology is divided into three stages: First, important financial performance measure of Saudi banks will be obtained. This will be followed by a step-wise regression analysis to establish the main contributors of the stability of Saudi banks. Lastly, ARIMA and exponential smoothing models will be chosen to make *Z*-score projections up to 2030.

Table 1 presents the operational definitions of the variables to be used in the current study. Banking stability is the dependent variable, and the Z -score (Z Score) is a measure that is widely used in the banking literature to capture the distance of insolvency, as a wide-scale indicator of the financial stability. The *Z*-score summarizes the cumulative effects of the profitability, leverage, and volatility, and as such, it provides a strong gauge of the stability of the banking institutions in general.

**Table 1:** Operational definitions.

	Variables	Variable label	Defined variable	Description
Dependent	Stability	*Z* Score	*Z* Score	$\frac{\mathrm{ROA}+\frac{\text{Equity}}{\text{Assets}}}{\partial \mathrm{ROA}}$
Independent	Banks’ wealth	AssetsLn	Bank size	*ln of Total Assets*
Independent	Asset quality	LLTE	Loan loss to total equity	$\frac{\text{Loans Imapairment}}{\text{Total Equity}}$
Independent	Liquidity	InvTA	Investment to total assets	$\frac{\text{Investments}}{\text{Total Assets}}$
Independent	Profitability	NII1	Net interest income ratio	$\frac{\mathrm{Net}\;\text{Interest Income}}{\text{Total Assets}}$
Independent	Capitalization	CAR	Capital adequacy ratio	$\frac{\text{Tier}\;1}{\text{Risky Weighted Assets}}$
Independent	Economic growth	GDP	GDP growth rate	*GDP*

The independent variables include significant bank-specific and macro-economic factors of stability. The size of the banks is operationalized by the natural logarithm of total assets (AssetsLn) which is the measure of both the wealth and the scale of the operations of the banks. The quality of assets is expressed by the loanloss to total equity ratio (LLTE) thus showing exposure to credit risk. The ratio of investments to total assets (InvTA) is used as a proxy of liquidity, indicating the degree of liquid assets. The measure of profitability is through the net interest income ratio (NII1) whereas the measure of capitalization is the capital adequacy ratio (CAR), which portrays the resilience of banks against the financial standards and regulatory requirements. Moreover, the economic growth is quantified in terms of gross domestic product growth rate (GDP), which is one of the macroeconomic control variables to consider the impact of overall economic conditions on the stability of banking.

### 3.2 Forecasting *Z*-score using ARIMA models

In this step, the study aims to forecast the *Z*-score for banking stability, for eleven major Saudi banks through 2030 In order to do this, we use time series forecasting techniques, in particular ARIMA (AutoRegressive Integrated Moving Average) models combined with different exponential smoothing techniques, each calibrated to particular characteristics we have observed in the Z score trajectories of individual banks. The process of modeling and forecasting is implemented using IBM’s application software package, namely, IBM’s statistical package (SPSS) Expert Modeler, which offers the function of automatic model selection of the most suitable model depending on the properties of the data and the fitness rules.

The ARIMA (AutoRegressive Integrated Moving Average) model is widely considered to be part of economic and financial time series forecasting for being flexible to handle both stationary and non-stationary series. It detects autocorrelation that exists in historical observations that helps to make correct short to medium predictions. In cases where a time series has a trend and seasonality, the Expert Modeler module in the software program, Statistical Package and Systems (SPSS), might instead suggest exponential smoothing methods, for instance Holt’s Linear Trend or Simple Exponential Smoothing. These models are proven to be beneficial in scenarios where the data have a linear trend or are relatively stable over time.

#### 3.2.1 Model fitting and validation

To test the reliability and robustness of the forecasts, the models selected for this study were tested with several goodness-of-fit indicators. These metrics informed the selection of the model that balances the need for parsimony with the accuracy of the model for each bank, as described below:

- *E-square (Error Sum of Squares)*: measures the total squared deviation between observed and predicted values.
- *RMSE (Root Mean Square Error)*: indicates the standard deviation of residuals, offering insights into the model’s predictive accuracy.
- *MAPE (Mean Absolute Percentage Error)*: provides the average percentage error, useful for interpretability in practical settings.
- *MAE (Mean Absolute Error)*: measures average absolute difference between observed and predicted values.

#### 3.2.2 Forecasting horizon and implementation

This modeling phase aims at developing projections for *Z*-scores for banks up to the year 2030, in order to explain the expected performance of the banking stability in Saudi Arabia. The forecasting process takes years of time and involves strict validation procedures aimed at ensuring the fulfillment of important criteria such as the reduction of residual autocorrelation, the avoidance of overfitting, and the stability of parameters. In the process of forecast generation, re-estimation of forecasts is performed during each iterative step in an adaptive modeling framework that dynamically modulates forecasts. This methodology adds model flexibility and reduces the progressive build-up of forecast errors. Standardized *Z*-score measures among all banking institutions are used to compare models and eliminate the effects of heteroscedasticity. The standardization procedure defines a uniform scale and enables therefore more precise performance evaluation of models and institutions of different levels of hierarchy. The methodology takes a model-specific approach to the unique characteristics of banking data and in doing so produces accurate long term *Z*-score forecasts, which are individually calibrated for each financial institution. Projections based on this methodology are aimed at helping stakeholders to implement data driven decisions to increase the sustainability of the Saudi banking sector while the country develops towards the goals outlined in Vision 2030.

## 4 Analysis and results discussion

### 4.1 Data analysis for independent and dependent variables

Table 2 provides crucial statistical information about the data set for analyzing Saudi banking stability as displayed in Table 2. A total of 87 observations featured the seven stability factors which included Capital Adequacy Ratio (CART1), Loan Loss to Total Equity (LLTE), Net Interest Income to Total Assets (NII1), Natural Log of Total Assets (AssetsLn), Investment to Total Assets Ratio (InvTA), GDP Growth Rate (GDP), and the *Z*-score. These statistical data provide understandings of regular patterns and distribution along with extreme points of each influence factor for banking performance in Saudi Arabia.

**Table 2:** Descriptive statistics.

Variable	*N*	Minimum	Maximum	Mean	Std. deviation
CART1	87	14.05	27.48	19.6300	2.37137
LLTE	87	2.12	22.05	10.6017	4.17635
NII1	87	1.46	4.28	2.6597	0.46662
AssetsLn	87	10.7	13.7	12.074	0.6415
InvTA	87	5.76	35.36	20.1348	7.25578
GDP	87	−4.34	4.69	1.8508	2.66550
*Z*-score	87	10.00	64.00	24.9317	15.54960

The calculated mean Capital Adequacy Ratio (CART1) value of 19.63 shows that the participating banks exceeding international regulatory capital requirements. The Capital Adequacy Ratio values ranging from 14.05 to 27.48 indicate all banks follow capital adequacy requirements yet some institutions operate with substantial buffer capacity. Capital adequacy measurements in the Saudi Arabian banking industry display modest variability because the standard deviation stands at 2.37.

The Loan Loss to Total Equity ratio (LLTE) demonstrates wider variation than other risk measurement indicators because it measures bank credit risk exposure. The variable indicates meaningful Loan Loss to Total Equity variation among financial institutions because it presents a mean of 10.60 and a standard deviation of 4.18. The extreme variation between maximum and minimum recorded Loan Loss to Total Equity figures demonstrates banks have significantly different approaches to reducing credit risk with the top value at 22.05 and the lowest at 2.12.

The NII1 measurement shows uniform profitability in banks through its average value of 2.66 and narrow dispersion of 0.47 standard deviations. A bank needs this metric to evaluate its effectiveness in producing profits from its total assets. Most banks maintain operation within a profitable zone which ranges between 1.46 and 4.28 due to standard business practices and interest rate conventions common throughout the sector.

The calculation of assetsLn through the natural logarithm yields an average bank size of 12.07 in logarithmic units. Bank size data show some variation based on the distribution between 10.7 to 13.7 while standard deviation at 0.64 maintains that size differences are not too extreme. A log transformation of data aids in reducing non-normal distribution patterns in size measurements while preparing the data for regression and time-series analysis.

The Investment to Total Assets Ratio (InvTA) displays the most extensive fluctuation and the highest variation at 7.26. The investment ratios across the banks are measured as 20.13 while the minimum value stands at 5.76 and the maximum at 35.36. Banks have varying degrees of asset allocation risk tolerance depending on strategic differences inherent in their core investment paradigms. The heterogeneity in investment strategies of the financial institutions can be attributed to the fact that some of the banks prefer to invest in more liquid and less riskier assets and some try to achieve higher returns by investing in riskier investment ventures.

The GDP growth rate forms part of the macroeconomic indicators which is important to know the conditions that influence the banking operations at the country level. A value of 1.85 per cent is taken to imply moderate economic expansion; while the reduction to −4.34 per cent is indicative of a recession that may be caused either by a shock to oil prices or by general financial turbulence in the world economy. The magnitude of economic volatility as measured by a standard deviation less than 2.67 has a substantial impact on financial outcomes. The *Z*-score as the main analytical indicator is highly variable over the study period. Distributional analysis shows scores between 10.00 and 64.00 with mean = 24.93 and SD = 15.55. This high dispersion suggests that there are significant differences in insolvency risk at the individual bank level.

The existence of non-standard safety buffers between different institutions also underscores the need for specific risk evaluations and regulatory intervention. Descriptive statistical synthesis suggests that banking institutions have strong capital structures and show profitable functioning performance, while at the same time, they show heterogeneous profiles of vulnerability, diverse investment strategies and levels of operational stability. Multiple banking institutions require distinct models which provide the basis for employing ARIMA forecasting techniques.

### 4.2 Correlation analysis

The correlation analysis in Table 3 shows the key attributes that influence banking stability in Saudi Arabia as measured by the *Z*-score. Financial institutions having higher capital reserves (CART1) demonstrate enhanced stability according to the data exploration results. Better financial resources enable a bank to survive adverse economic conditions better. The measured positive and statistically significant link confirms that banks need substantial capital reserves as a vital factor for their overall stability. Our data shows that the opposite effects happen when we study loan loss to total equity (LLTE). The strong negative association between *Z*-score stands logical since the findings are expected. Bad loans that exceed loan losses reduce bank capital levels and drove investors to lose confidence in the institution. The increase in credit risk automatically decreases operating stability for banking institutions. The empirical data point that risk management is a material reality that defines the resilience of banks.

**Table 3:** Correlation matrix.

Variable		*Z*-score	CART1	LLTE	NII1	AssetsLn	InvTA	GDP
*Z*-score	Pearson Correlation	1.00						
	Sig. (2-tailed)							
CART1	Pearson Correlation	0.422^*^	1.00					
	Sig. (2-tailed)	0.05						
LLTE	Pearson Correlation	−0.377^**^	−0.11	1.00				
	Sig. (2-tailed)	0.00	0.32					
NII1	Pearson Correlation	0.328^*^	0.19	0.353^**^	1.00			
	Sig. (2-tailed)	0.04	0.08	0.00				
AssetsLn	Pearson Correlation	0.722^*^	0.00	−0.17	0.361^**^	1.00		
	Sig. (2-tailed)	0.02	0.98	0.11	0.00			
InvTA	Pearson Correlation	−0.18	0.217^*^	−0.16	−0.321^**^	0.20	1.00	
	Sig. (2-tailed)	0.09	0.04	0.13	0.00	0.06		
GDP	Pearson Correlation	0.08	−0.243^*^	−0.246^*^	−0.231^*^	−0.02	−0.14	1.00
	Sig. (2-tailed)	0.48	0.02	0.02	0.03	0.87	0.21	

**Correlation is significant at the 0.01 level (2-tailed). *Correlation is significant at the 0.05 level (2-tailed).

Stability had a statistically significant positive relationship with bank profitability, as measured by net interest income (NII). Revenue from lending activities reflects efficient banking business, thus increasing the ability to face unforeseen losses. The earning of profits is used for the benefit of investor payouts and also serves as a financial buffer for banks in adverse situations. Business assets (AssetsLn) were established among the highest links with the *Z*-score metric. Due to their larger size and diverse product lines banks gain stability advantages.

A minimal negative pattern emerged between stability and investment to total assets (InvTA) yet it did not deliver enough evidence to draw final conclusions. High investment levels particularly when centred on risky assets might diminish bank stability. The connection between GDP growth and *Z*-score proved to be underwhelming because the results were both weak and statistically unimportant. The lack of correlation between economic prosperity and banking stability surprised because rising economies traditionally benefit their banking institutions. Several unknown elements probably contribute to this situation. Our analysis verifies basic principles about banking stability which consolidate capital strength with minimal losses and strong earnings and enlarged size. These basic analysis results fail to support a strong relationship between investment ratios and GDP but this does not rule out their significance within more advanced mathematical models.

### 4.3 *Z*-score based on linear regression model

For brevity, only the best model of the stepwise models is reported as shown in Table 4. The stepwise regression analysis identified Model 5 because it represented the most effective fit for understanding variations in the *Z*-score which functions as the stability measure for Saudi banking institutions. Among the tested five models Model 5 showed the most appropriate fit. The selected model utilizes Capital Adequacy Ratio (CART1), Bank Size (AssetsLn), Loan Loss to Total Equity (LLTE), Net Interest Income (NII1), and Investment to Total Assets (InvTA) as its main predictive variables. The five included variables effectively explain 71.3% of *Z*-score variations according to the adjusted R^2^ value of 0.713. The model demonstrates a powerful fit because it accounts for most factors which affect banking stability. The 7.21 standard error indicates a precise prediction for this model based on the low level of expected errors. The analysis included a Durbin-Watson statistic to ensure that the residuals (errors) are randomly distributed, ensuring a prerequisite for a robust regression model. The autocorrelation of residues or errors results which is 1.778 is in the acceptable range 1.5–2.5, which indicates no significant bias.

**Table 4:** Model summary.

Model	*R*	*R* square	Adjusted *R* square	Std. error of the estimate	Durbin-Watson
5	0.856^a^	0.732	0.713	7.20896	1.778

a Predictors: (Constant), CART1, AssetsLn, LLTE, NII1, InvTA.

The *F* statistic is statistically significant, at the 1 percent level, and thus confirms that the entire regression model provides substantive results that are beyond random variation. The stability predictors show strong importance for Saudi banks while the developed model presents a strong base for future predictive work.

Table 5 shows that the regression model is statistically significant, with an *F*-value of 38.837 and a *p*-value of 0.000, confirming the model’s overall validity. The large regression sum of squares (10,091.62) compared to the residual (3,689.81) indicates the model explains most of the variance in *Z*-score. This supports the strong predictive power of the selected variables.

**Table 5:** ANOVA.

Model		Sum of squares	Df	Mean square	*F*	Sig.
5	Regression	10091.62	5	2018.324	38.837	.000^b^
	Residual	3689.808	71	51.969		
	Total	13781.429	76			

b Predictors: (Constant), CART1, AssetsLn, LLTE, NII1, InvTA.

A VIF (Variance Inflation Factor) filtering stepwise regression method was utilized for independent variable multicollinearity assessment based on the earlier correlation matrix results. The model includes all variables with VIF scores lower than 10 to prevent significant multicollinearity in the final analysis. Model 5 required extensive analysis because of its five very robust indicators including Capital Adequacy (CART1), Bank Size (AssetsLn), Asset Quality (LLTE), Profitability (NII1) and Investment Ratio (InvTA) that provided significant contributions in *z*-score evaluation. The VIF values included in Table 6 stay below 10 thus confirming that the variables exhibit minimal to no multicollinearity issues. The t-values and *p*-values confirm the entire set of predictors that influence banking stability with significance at levels reaching or below 5%. The results indicate AssetsLn serves as the most influential bank factor (Beta = 0.571, *p* < 0.001) because of its impact on financial resilience. The LLTE and InvTA variables produce negative results. This finding strengthens the idea that high credit risks and over-investments hurt the banking profitability. The model includes an intercept value of −81.656 to properly adjust the regression line in line with the exact effects of the included variables. This refined model provides statistical rigour and interpretability in terms of mitigation of multicollinearity that makes it suitable for forecasting banking stability and policy implications for Saudi Arabia. Profitability is a key factor in bank stability; high profits represent effective management performance and often are accompanied by a rise in the company’s stock price. Such an increase helps from the point of view of maximisation of wealth, which is a basic aspect of desired stability. In addition, an increase in bank revenue can increase profit margins in operating results, which can increase bank stability. Our analysis provides support for alternative hypothesis of the positive and significant effect of NII1 on the augmentation of bank stability, which is consistent with the findings of Ledhem and Mekidiche (2020), Peura and Keppo (2006), Sun et al. (2017), and Elsayed et al. (2023), implying the importance of profitability in promoting stability. Liquidity refers to the ability of a bank to meet short-term financial commitments out of short-term assets. The effect on the stability of banks is an outcome of the fact that with an increase in total investment, a reduction in interest income gained, leading to a lower profit margin and, ultimately, a lower stability score. This result is consonant with the results of Sun et al. (2017), which highlight the adverse effect of liquidity on bank stability. Capitalisation measured by the CAR ratio has a positive impact on bank stability. A high CAR implies a better level of efficiency and stability, as it reduces the risk of insolvency and the fulfillment of financial obligations. A high CAR ratio indicates that Islamic banks possess sufficient capital for their risk profile, and hence stability. Our results show a positive and significant effect of the capital ratios on the stability of banks, which is in line with the studies of Ghassan and Taher (2013), Ledhem and Mekidiche (2020), Karim et al. (2018), Albaity et al. (2021), Beck et al. (2013), Kharabsheh and Gharaibeh (2022), Kamran et al. (2019), and Daoud and Kammoun (2020).

**Table 6:** Linear regression model coefficients.

Model		Unstandardized		Standardized	*t*	Sig.	Collinearity	
		Coefficients^a^		Coefficients^a^			Statistics	
		*B*	Std. Error	Beta			Tolerance	VIF
5	(Constant)	−81.656	21.064		−3.876	0		
	CART1	1.991	0.589	0.218	3.382	0	0.925	1.082
	AssetsLn	12.522	2.114	0.571	5.922	0	0.406	2.465
	LLTE	−1.143	0.231	−0.357	−4.938	0	0.72	1.389
	NII1	5.978	2.429	0.192	2.461	0.016	0.617	1.62
	InvTA	−0.307	0.135	−0.163	−2.267	0.026	0.733	1.365

a Dependent variable: *Z*-score.

The quality of assets and the provision of loans in banking institutions has received a lot of scholarly interest considering the fact that loans form a substantial portion of asset portfolio of banks. When assets are highly risky, their value in the market can depreciate precipitously. Asset quality therefore largely concerns the management of the loan portfolio by the banks to maximize income and minimize the case of non-performing loans. Consequently, non-performing financing has the potential to affect the financial position and the overall stability of the banking sector. Our empirical findings show a significant negative relationship between the higher loan losses and bank stability corroborate the results reported by Kumar (2022), Suljić Nikola et al., 2022, Chai et al. (2022), Maritsa and Widarjono (2021), Adusei (2015), Ghenimi et al. (2017), Zaghdoudi (2019), Kharabsheh and Gharaibeh (2022), Prima Sakti and Mohamad (2018), and Ajizah and Widarjono (2023). Lastly, moving to the proxy of banks’ wealth, a bank that is larger can better withstand financial shocks. Banks that are wealthy can leverage their larger asset base to distribute their fixed costs, resulting in reduced average expenses and increased profits through the advantages of economies of scale. This suggests that higher levels of a bank’s wealth can enhance the stability of its performance. Our analysis supports the alternative hypothesis that AssetsLn has a positive and significant impact on increasing bank stability, which is consistent with Regehr and Sengupta (2016), Widarjono (2020), Ajizah and Widarjono (2023), Rajhi and Hassairi (2013), Beck (2008), Slimen et al. (2021), Rahim et al. (2012), and Čihák and Hesse (2010).

Lastly, the lack of a significant effect of GDP could be explained by the following factors on several structural pillars of the Saudi economy:

- Oil-Based Economy: Saudi GDP is highly susceptible to changes in the global oil prices as it is a major contributor of the governmental revenues compared to actual banking operations.
- Regulatory Framework: Saudi banks are regulated under strict regulatory policies of the Saudi central bank (SAMA) such as well-developed capital adequacy and liquidity requirements. This form of regulatory strength provides immunity against microeconomic fluctuations of short-term nature.
- Government Support and Vision 2030 Programs: Indirectly, the Saudi government assists the banking sector by high level of investment programmes, which in turn ensures that the banks are not heavily dependent on economic growth in the short run.
- Growing Banking Revenue Bases: Saudi financial institutions make revenues through strong retail deposits, lending related to governments and Islamic financing model, which are not very sensitive to the changes in GDP. The economic growth, although theoretically predicted to lead to stability, is not statistically significant in explaining stability in Saudi banks within the period considered in the present study.

### 4.4 Forecasting *Z*-score using ARIMA models

In order to strengthen the model-selection process as well as make it more transparent, formal information criteria were included in the forecasting framework. In particular, the Akaike Information Criterion (AIC) and Bayesian Information Criterion (BIC) were used as the main diagnostics alongside the forecast-accuracy metrics, i. e. RMSE, MAE, MAPE, the squared error (E -square). These criteria ensure that there is optimum trade-off between the model fit and parsimony penalizing unnecessary complexity.

The AIC places great importance on in-sample goodness of fit with a moderate penalty on the number of parameters estimated. The BIC on the other hand is more strict in over-parameterisation and is especially suitable when the sample is small and medium-sized. As part of the estimation process of each banking institution, a variety of ARIMA and exponential-smoothing models were estimated using IBM SPSS Expert Modeler; the best model was selected based on the lowest combined AIC and BIC values, subject to reasonable residual diagnostics and forecast-accuracy statistics. This two criterion model is helpful in reducing overfitting and providing statistically efficient forecasts in the long run.

The chosen forecasting models for *Z*-score prediction, used as a banking stability metric, serve as a summary for eleven major Saudi banks, as shown in Table 7. SPSS categorized the selected forecasting models by analyzing bank data time series and then determined the most suitable approach according to statistical criteria.

**Table 7:** Model description for all the banks.

Bank name	Model type
Al Rajhi Bank (RJHI AB)—Standardized	ARIMA (0, 0, 0)
Alinma Bank (ALINMA AB)—Standardized	ARIMA (0, 1, 0)
Arab National Bank (ARNB AB)—Standardized	Holt
Bank Al-Jazira (BJAZ AB)—Standardized	Simple
Bank AlBilad (ALBI AB)—Standardized	Holt
Banque Saudi Fransi (BSFR AB)—Standardized	Holt
National Commercial Bank (NCB AB)—Standardized	ARIMA (0, 0, 0)
Riyad Bank (RIBL AB)—Standardized	ARIMA (0, 1, 0)
Samba Financial Group (SAMBA AB)—Standardized	ARIMA (0, 0, 0)
Saudi British Bank/The (SABB AB)—Standardized	ARIMA (0, 0, 0)
Saudi Investment Bank/The (SIBC AB)—Standardized	ARIMA (0, 0, 0)

Here’s what the table reveals:

- The ARIMA (0,0,0) specification was best fitted to several banks, that is, Al Rajhi Bank, National Commercial Bank, Samba Financial Group, Saudi British Bank, and Saudi investment Bank. ARIMA (0, 0, 0) statistically refers to a white-noise stationary process with constant mean, which does not contain significant autoregressive, moving-average or differencing components. This modeling result suggests that the series of *Z*-score of these banks has: high stochastic stability, no leading trend or seasonal structure, and poor time dependence. Economically and financially balanced the result is a solid internal risk management, long-term capital sufficiency, stable profitability and diversified revenue formations. Therefore, the choice of ARIMA (0,0,0) to these institutions does not imply modeling weakness, but it proves the financial stability of the structure as well as the ability to withstand the shocks, which depend on time. More complicated ARIMA options were also tried but repeatedly provided more high AIC and BIC, which expressed low explanatory power.
- The best forecasting structure found in the case of Bank Al-Jazira was the Simple Exponential Smoothing (SES). SES is a theoretically suitable method under the following conditions in the time series: Exhibits no significant time trend, Is seasonal, Fluctuates about a fixed mean, and Is filled with short-periodic random disturbances. The trend-based exponential smoothing and ARIMA alternatives all gave larger values of AIC and BIC, which means that they are over-parameterized and have less predictive power. Thus, the choice of SES was made with statistical optimality and structural consistency of *Z*-score dynamics, without regard to simple models.
- In case of Alinma bank and Riyad bank, ARIMA(0,1,0) was the best form because it had non-stationary trend, which meant that first differencing had to be done to attain statistical stationarity. Such an outcome is an indication of structural variation of risk exposure and financial performance, reinforcing the stability negative variations of the trajectories.
- The Linear Trend Model created by Holt was also chosen in Arab National Bank, Banque Saudi Fransi, and Bank AlBilad due to the fact that their series of *Z*-score shows consistent linear trends. Competing ARIMA specifications were inferior, in terms of lower AIC and BIC values, to the model created by Holt, and showed the existence of economically significant deterministic trend components.

Various banks demonstrate unique stability characteristics because their *Z*-scores follow different paths according to the data presented. Different financial stability patterns need specialized forecasting approaches which span from basic ARIMA models to trend-sensitive exponential smoothing methods for producing reliable long-term predictions of stability.

Based on the analysis of the model parameters in Tables 8, 9, the ARIMA model demonstrates superior performance compared to the Exponential Smoothing model. In Table 8, which summarizes the ARIMA model parameters, constant (drift) terms of Alinma Bank and Riyad Bank were statistically insignificant (*p* > 0.05). This means that there is no significant dynamic behavior of systematic patterns in the differenced series. This specification is in effect a random walk without prediction or economic explanation. The ARIMA results were therefore not trusted and no other advanced analysis was conducted on both the banks to ensure the strength of the empirical results. Several banks such as Al Rajhi Bank, National Commercial Bank, Samba Financial Group, Saudi British Bank, and Saudi Investment Bank show extremely high and statistically significant constant estimates with very low standard errors and highly significant t-values (all with Sig. = 0.000). Notably, Saudi Investment Bank shows the strongest result with a *t*-value of 46.378, indicating very strong statistical significance and stability in the model. In contrast, Table 9, which presents the Exponential Smoothing model parameters, reveals much weaker and less statistically significant results. For example, Arab National Bank and Banque Saudi Fransi both have non-significant alpha (level) parameters, with *p*-values of 0.441 and 0.675, respectively. Additionally, the Gamma (trend) parameters across the board are extremely small and non-significant (*p*-values = 1.000), indicating that the model failed to capture meaningful trends in the data for most banks. Only Bank Al-Jazira has a somewhat significant alpha estimate (*p* = 0.027), but this is isolated and not consistent across the dataset. Research evidence shows that the ARIMA model produced superior outcomes by demonstrating high explanatory variables with significant statistical values in banks including Saudi Investment Bank and National Commercial Bank. The ARIMA model offers a better framework for evaluating *Z*-scores throughout Saudi banks because it demonstrates enhanced reliability and robustness.

**Table 8:** ARIMA model parameters.

Bank name	Estimate	SE	*t*	Sig.
Al Rajhi Bank (RJHI AB)—Standardized constant	13.640	0.680	20.052	0.000
National Commercial Bank (NCB AB)—Standardized constant	36.635	1.610	22.754	0.000
Samba Financial Group (SAMBA AB)—Standardized constant	27.294	1.434	19.035	0.000
Saudi British Bank/The (SABB AB)—Standardized constant	19.826	0.700	28.332	0.000
Saudi Investment Bank/The (SIBC AB)—Standardized constant	17.908	0.386	46.378	0.000

**Table 9:** Exponential smoothing model parameters.

Bank Name	Estimate		SE	*t*	Sig.
Arab National Bank (ARNB AB)—Standardized	Alpha (Level)	0.198	0.239	0.825	0.441
Bank Al-Jazira (BJAZ AB)—Standardized	Gamma (Trend)	2.360E-06	0.219	1.080E-05	1.000
	Alpha (Level)	1.000	0.359	2.787	0.027
Bank AlBilad (ALBI AB)—Standardized	Alpha (Level)	0.001	0.590	0.002	0.999
	Gamma (Trend)	0.000	332.670	8.500E-07	1.000
Banque Saudi Fransi (BSFR AB)—Standardized	Alpha (Level)	0.099	0.226	0.441	0.675
	Gamma (Trend)	3.062E-05	0.909	3.368E-05	1.000

**Figure 1:**
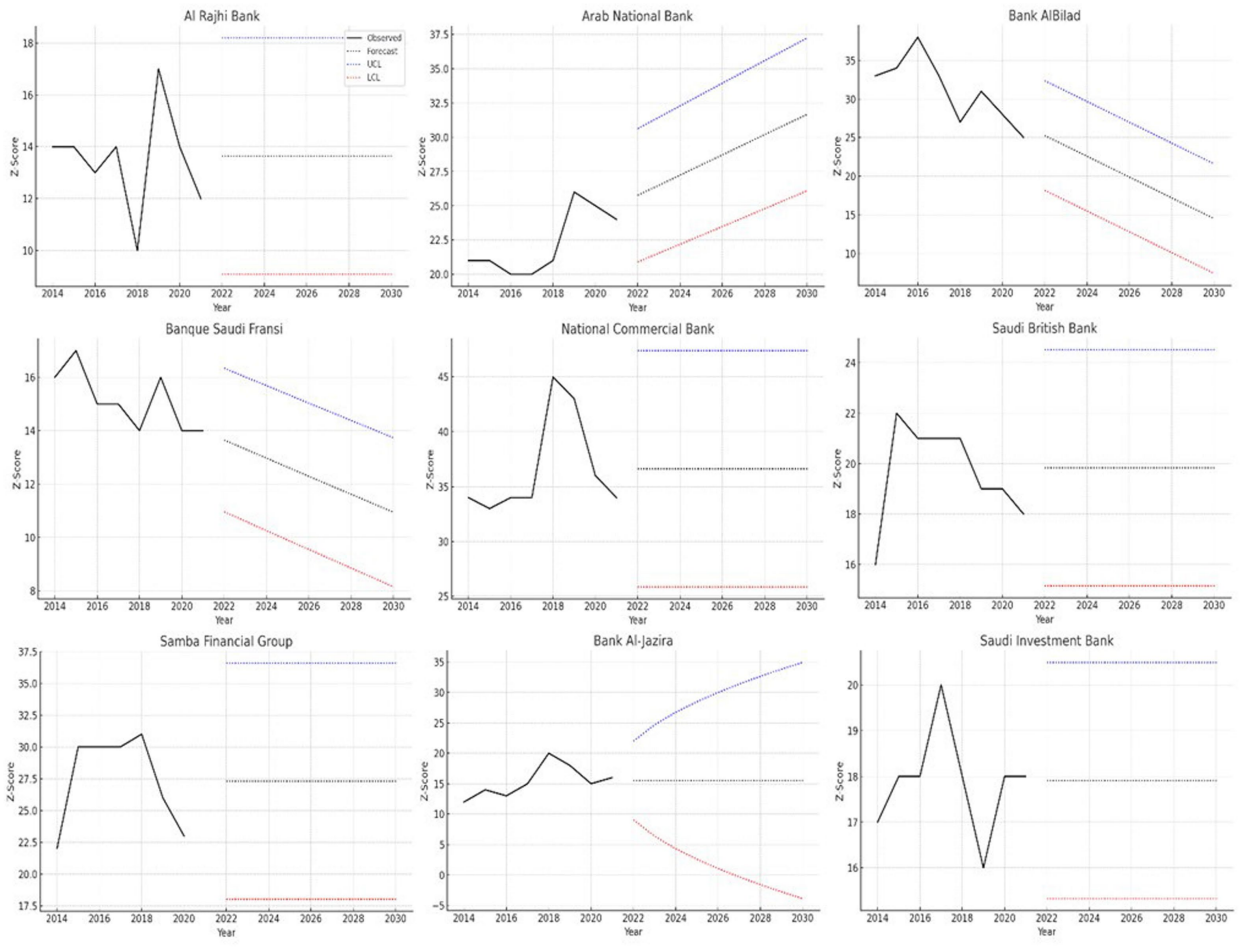
Forecasting for *Z*-score value of the banks.

Figure 1 depicts the past and future trends of the *Z*-score of nine Saudi banks between the years 2014 to 2030, along with the upper and lower confidence limits as well. The *Z*-score is a proven measure of the risk of insolvency, with high values indicating more financial healthiness. The figure shows obvious heterogeneity in the dynamics of stability of Saudi banks.

A group of the first banks such as Al Rajhi Bank, National Commercial Bank, Saudi British Bank, Samba Financial Group, and Saudi Investment Bank is characterized by *Z*-score paths which are most stable both in the historical and in the forecast horizon. Their projections take almost horizontal directions and this agrees with the ARIMA (0,0,0) specifications adopted in these institutions. This trend validates their high structural stability, continued capital buffers, diversified sources of income, and low cyclical risk exposure, suggest long-term financial resilience until 2030.

Another second category of banks, which are Arab National Bank, Banque Saudi Fransi and Bank AlBilad, exhibits trend-oriented dynamics, as are the trend models of the Holt linear trend models. These banks remain at medium levels of stability but show slow decreasing curves, which reflect progressive strain towards the financial soundness of the long-run instead of sudden disturbance.

Lastly, Bank Al-Jazira pursues a unique trend that is gradually rising, which is best represented by the Simple Exponential Smoothing model. Its forecasted *Z*-score increases continuously up to 2030 with close confidence intervals, as an indicator of gradual reinforcement of its stability status.

In general, Figure 1 shows that the future banking stability in Saudi Arabia does not take a uniform trend among institutions. Even though large and systemically important banks maintain good resilience, multiple medium-sized banks exhibit the increasing financial risk. These results give early signals to regulators and policymakers, allowing them to intervene in supervision before problems arise and capital and liquidity reinforcement to protect the banking system as part of Vision 2030.

The financial stability results in Figure 1 demonstrate notable differences in the Saudi banks between 2014 and 2030 when using both actual and projected *Z*-scores. The *Z*-score serves as a well-known risk assessment tool for insolvency because stronger financial positions yield higher scores but weaker positions generate lower scores. Real data together with projected values and Upper Control Limit (UCL) and Lower Control Limit (LCL) establish a comprehensive understanding of bank historical performance and forecasted evolution. The financial performance of Arab National Bank together with National Commercial Bank shows steady trends throughout the observation period. The banks maintain a stable predicted *Z*-score performance between 2030 and their predicted LCLs stay above the Banking authorities project these financial institutions to preserve minimal financial risk as a permanent condition. Saudi Investment Bank as well as Bank Al-Jazira demonstrate predictable behavior through their moderate consistent performance metrics which are supported by tight confidence interval ranges. A number of banking institutions exhibit either cautious or unchanging projection patterns. The financial forecast patterns at Al Rajhi Bank together with Samba Financial Group along with Saudi British Bank represent flat expectations for financial performance through upcoming years.

The fundamental reasons include a highly diversified income base, retail banking predominance, high low-cost deposit base, tight lending policy, and enduring capital surpluses that are beyond regulatory levels. These factors serve as natural shock absorbers to macroeconomic and financial shocks hence explaining the witnessed stability in the regression analysis and forecasting stages. The results are quite in line with the works of Kharabsheh and Gharaibeh (2022) and Mabkhot and Al-Wesabi (2022), as they prove that the impact of capitalization and asset quality has a stronger effect than that of macroeconomic variables on the stability of the bank. Moreover, it is stated that growing asset aggressively and high financing concentration correlate with a decrease in the *Z*-score by Ajizah and Widarjono (2023) and Rahim et al. (2012). Also, Chai et al. (2022) and Ghenimi et al. (2017) affirm that credit and liquidity risk have more significant impacts on stability compared to GDP growth, which supports our findings. Therefore, the findings obtained in the case of Saudi Arabia are not unique to the country; instead, they capture the wider region and the emerging-market banking trends.

The space between confidence interval lines provides valuable information about the analysis. The forecast precision and risk volatility of Saudi Investment Bank and Bank Al-Jazira is high as their UCL-LCL interval remains limited. Multiple banks are forecast to maintain good results, but a portion of banking entities will possibly suffer in the event of continued negative financial trends. Predictive data serves as an important tool for stakeholders to assess financial operations, which will aid in funding decisions and help regulators maintain their oversight.

## 5 Conclusions and implications

The main objective of this paper is to find out the key determinants that affect the stability of banks in Saudi Arabia. To achieve this aim, a stepwise linear regression analysis was adopted using the financial data included in the annual reports of eleven active Saudi banks from 2014 to 2021. The explanatory variables were clustered under six categories: wealth, quality of assets, liquidity, profitability, capitalization and economic growth. The stability Z-scor was defined as dependent variable and selected performance indicators were used as independent variables. Model selection was based on 5 candidate specifications that had the highest adjusted *R*-squared values and smallest values of standard errors.

Empirical results show that bank stability is significantly and positively related to profitability (as measured by the net interest income to total income (NII1) ratio), wealth (as measured by the logarithm of total assets) and capitalization (as measured by the capital adequacy ratio (CAR)). In contrast, liquidity (investment ratio) and asset quality (loans impairment ratio) have a significant negative impact on the stability *Z*-score. The growth of the national economy, which is proxied by the gross domestic product (GDP), was found to be statistically insignificant in terms of predicting bank stability. These results are broadly consistent with the literature that exists, except for the economic growth variable.

In addition to the explanatory framework, this study includes a forecasting dimension, based on time-series techniques. Autoregressive integrated moving average (ARIMA) models and exponential smoothing methods were used to project the *Z*-score of each bank up through the year 2030. The best specifications were chosen using the statistical software package IBM SPSS Expert Modeler and then tested against performance measures such as root mean squared error (RMSE), mean absolute error (MAE), mean absolute percentage error (MAPE) and the squared correlation coefficient (E-square). When normalized for comparison purposes, the forecasts reveal heterogeneous trends for future risk in the banking sector where some institutions, i.e., Arab National Bank, Al Rajhi Bank, are expected to maintain stable risk profiles while others are expected to face notable risk degradation. The addition of a forecast dimension adds to the empirical importance of the present investigation as it allows relevant stakeholders to forecast future stability outcomes and to adjust their action accordingly.

The results of this investigation have a considerable theoretical and empirical importance for researchers, shareholders and policy makers. Senior banking executives should adopt efficient and effective operational practices to strengthen the stability of their institutions to an acceptable institutional threshold. Additionally, bank leaders should carefully observe key stability indicators, especially capital adequacy ratio (CAR), non-performing loan (NPL) ratios, and investment ratios and to use specific strategies to improve the overall stability by improving the credit risk management, effective cost management, and revenue management. Bank executives should focus on creating additional revenue simultaneously with cost reduction, promoting wealth maximization for equity holders and, through extension to stability of the banking sector.

It is plausible that existing and prospective shareholders will benefit from this study by infusing investment capital into the equities of Saudi banks that are expected to display higher levels of stability. The predicted indicators focus on banks with dependable long-term trends, hence making investment decisions that are aligned to risk appetites. This study contributes to the overall stability of banks, strengthens the strategies of supervision, and supports the promotion of strategic investments, thereby enhancing the sustainability of the Saudi banking industry, in accordance with the Vision 2030.

Furthermore, policymakers in the Saudi Central Bank and regulatory institutions can use the findings of this study to conduct regular assessments of banks’ stability indexes and implement forward-looking supervisory strategies. These lessons can be institutionalized by the regulators like the Saudi Central Bank by having forward looking stress tests, scenario tests and systematic monitoring of the at-risk banks; they can therefore implement remedial instruments like raising capital buffers or providing liquidity support where it is required.

## 6 Limitations, and future studies

Despite the strength of the empirical model and the suitability of the used econometric and forecasting models, this research has some limitations which should be mentioned.

First, the study is conducted on a rather small sample of 11 Saudi banks between 2014 and 2021. As much as these banks are the central part of the Saudi banking industry, the scope of cross-sectional aspect is limited, which constrains the generalizability of the findings to smaller financial institutions or non-listed banks. In addition, the time horizon of 8 years is adequate to use regression and ARIMA modeling, but it may not be enough to represent all long-run structural cycle, especially the oil price super-cycle and the macro-financial regime shifts.

Second, the national commercial bank and samba financial group merger is a structural discontinuity of the dataset. The outcome of this merger is the loss of observations of Samba in 2021, which were interpolated statistically and seasonally adjusted. Although this is a sound methodology, it can still cause measurement distortion and smoothing bias during both the regression and forecasting process. The degree of structural change that could result in such vast changes in *Z*-score dynamics could not be entirely recreated by mere statistical modification.

Third, the research is based purely on secondary financial statements which are available as published annual reports. Similar to any other accounting-based research, the analysis can be prone to reporting delays, accounting discretion, and regulatory classification differences as this may influence the accuracy of certain financial ratios.

Fourth, the forecasting models make the assumption of structural consistency in the banking behavior and regulatory policy over the forecasting horizon until 2030. However, unexpected shocks, such as financial crises in the world, geopolitical conflicts, monetary tightening cycles, or regulatory changes, may alter the future stability patterns significantly and reduce the accuracy of long-term forecasts.

Lastly, the analysis focuses more on the bank-specific financial variables and GDP than other variables that may have been material in the analysis as they include inflation, interest-rate spreads, oil-price volatility, exchange-rate exposure, and market-competition indices. This may lead to omitted-variable bias in the regression model and prediction model since these variables are not included.

Future studies can resolve those limitations by expanding the temporal horizon, including more macroeconomic and market variables, and examining the dynamics after the mergers through structural-break models, and performing comparative research of the GCC countries to augment external validity. In addition, the latter studies may investigate which factors determine the stability of GCC banks. Lastly, one would be wise to use the indicators applied in the current study in alternative modeling tools, such as artificial neural networks and machine-learning methods, in order to increase the level of accuracy in forecasting and flexibility of the model.

## Data Availability

The raw data supporting the conclusions of this article will be made available by the authors, without undue reservation.
